# Metabolic Effects of Elicitors on the Biosynthesis of Tropane Alkaloids in Medicinal Plants

**DOI:** 10.3390/plants12173050

**Published:** 2023-08-24

**Authors:** Yuru Wen, Yiran Liao, Yueli Tang, Hongbo Zhang, Jiahui Zhang, Zhihua Liao

**Affiliations:** 1 Integrative Science Center of Germplasm Creation in Western China (Chongqing) Science City & Southwest University, School of Life Sciences, Southwest University, Chongqing 400715, China; yrwen66@outlook.com (Y.W.); yiranliao612@163.com (Y.L.); tangyueli_999@163.com (Y.T.); 2 SWU-TAAHC Medicinal Plant Joint R&D Centre, School of Life Sciences, Southwest University, Chongqing 400715, China; 3 Key Laboratory of Synthetic Biology of Ministry of Agriculture and Rural Affairs, Tobacco Research Institute of Chinese Academy of Agricultural Sciences, Qingdao 266101, China; zhanghongbo@caas.cn

**Keywords:** elicitation, hyoscyamine, medicinal plants, production, scopolamine, tropane alkaloids

## Abstract

Tropane alkaloids (TAs) are large secondary metabolite alkaloids that find extensive applications in the synthesis of antidotes, anesthetics, antiemetics, motion sickness drugs, and antispasmodics. The current production method primarily depends on extraction from medicinal plants of the Solanaceae family. Elicitation, as a highly effective biotechnological approach, offers significant advantages in augmenting the synthesis of secondary metabolites. The advantages include its simplicity of operation, low cost, and reduced risk of contamination. This review focuses on the impact of elicitation on the biosynthesis of TAs from three aspects: single-elicitor treatment, multiple-elicitor treatment, and the combination of elicitation strategy with other strategies. Some potential reasons are also proposed. Plant hormones and growth regulators, such as jasmonic acid (JA), salicylic acid (SA), and their derivatives, have been extensively employed in the separate elicitation processes. In recent years, novel elicitors represented by magnetic nanoparticles have emerged as significant factors in the investigation of yield enhancement in TAs. This approach shows promising potential for further development. The current utilization of multi-elicitor treatment is constrained, primarily relying on the combination of only two elicitors for induction. Some of these combinations have been found to exhibit synergistic amplification effects. However, the underlying molecular mechanism responsible for this phenomenon remains largely unknown. The literature concerning the integration of elicitation strategy with other strategies is limited, and several research gaps require further investigation. In conclusion, the impact of various elicitors on the accumulation of TAs is well-documented. However, further research is necessary to effectively implement elicitation strategies in commercial production. This includes the development of stable bioreactors, the elucidation of regulatory mechanisms, and the identification of more potent elicitors.

## 1. Introduction

**Figure 1:**
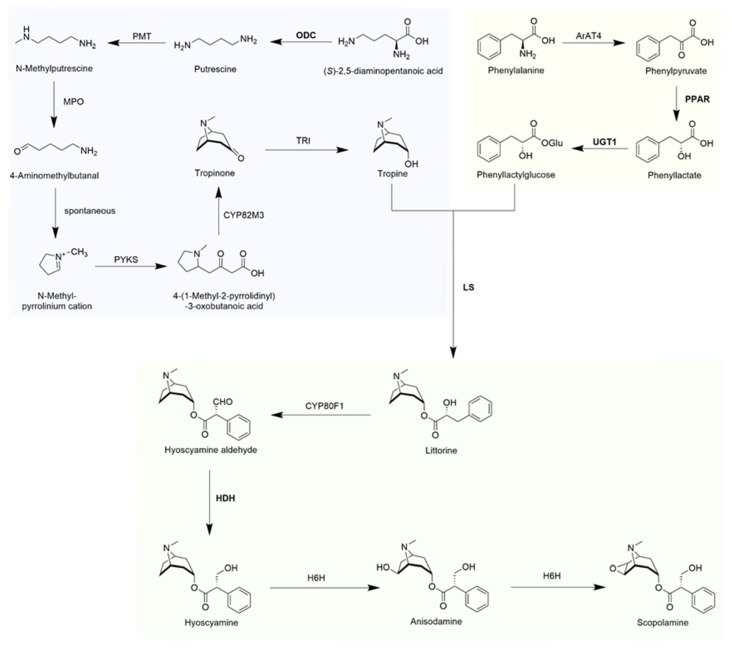
Synthetic pathways of medicinal tropane alkaloids in Solanaceae.

Tropane alkaloids (TAs) are a distinct class of alkaloids characterized by the presence of a tropane skeleton, which comprises a pyrrole ring and a piperidine ring. More than three hundred of them have been isolated and identified from plants of Solanaceae, Convolvulaceae, Proteaceae, Rhizophoraceae, etc., families [1,2]. The representative compounds hyoscyamine and scopolamine are of great interest as anticholinergic factors in the human parasympathetic nervous system. They have been used for the treatment of motion sickness, pesticide poisoning, Parkinson’s disease, anesthesia, analgesia, cough, and asthma relief [3]. The availability of hyoscyamine and scopolamine is still dependent on the isolation from a few Solanaceae family. However, the concentration of them in these plants is relatively low, with 0.2% dry weight (DW) and 0.02% DW in *Atropa belladonna*, an important medicinal plant for TAs [4]. This leads to many problems, such as a shortage of natural drug resources and high drug costs. Currently, the main strategies are chemical total synthesis or plant genetic engineering to enhance the content of natural products in medicinal plants, both of which rely on the complete resolution of the biosynthetic pathway of TAs [2,5] (Figure 1). Nevertheless, the total chemical synthesis of TAs has been hampered as a result of long synthetic routes, high-yield by-products, low yields, and high costs, making this approach less valuable for contemporary commercial applications. Due to the current statutory restrictions on the application of transgenic technologies, the direct commercialization of high-yield TAs transgenic plants produced through plant genetic engineering approaches is frequently challenging.

There are 13 TA biosynthetic genes in the synthetic pathway: ODC, ornithine decarboxylase; PMT, putrescine N-methyltransferase; MPO, N-methyl-putrescine oxidase; PYKS, pyrrolidine ketide synthase; CYP82M3, tropinone synthase; TRI, tropinone reductase I; ArAT4, aromatic amino acid aminotransferase; PPAR, phenylpyruvic acid reductase; UGT1, phenyllactate UDP-glycosyltransferase; LS, littorine synthase; CYP80F1, littorine mutase; HDH, hyoscyamine dehydrogenase; H6H, hyoscyamine 6β-hydroxylase.

Elicitation strategies are a technology that involves external interventions to directly stimulate the expression of target genes in cells or tissues, resulting in a significant enhancement of secondary metabolite production. This approach offers several advantages, including high efficiency, environmental friendliness, and low cost [6]. Elicitors that induce the immune response in plants do not need to participate in metabolic pathways. Instead, they function as signaling molecules that influence the biosynthesis and accumulation of secondary metabolites in plants. Numerous experiments have demonstrated that elicitation strategy can significantly enhance the production or accumulation of secondary metabolites in a variety of medicinal plant species. With further research on secondary metabolites and optimization of the elicitation strategy, this strategy holds promise for broader application and prospects.

We reviewed the effects of elicitors derived from different sources on the synthesis, accumulation, and release of TAs (especially hyoscyamine and scopolamine) in different Solanaceae plant species. Our analysis was based on an extensive review of the relevant literature. Additionally, we identified several current challenges and proposed future research directions for this strategy. It is imperative for researchers to comprehensively understand the recent advancements in the elicitation strategy for TAs synthesis to design and realize commercial applications. To our knowledge, this is the first material to provide a detailed review of the effects of various elicitors on the biosynthesis of TAs in medicinal plants.

## 2. Elicitors

**Figure 2:**
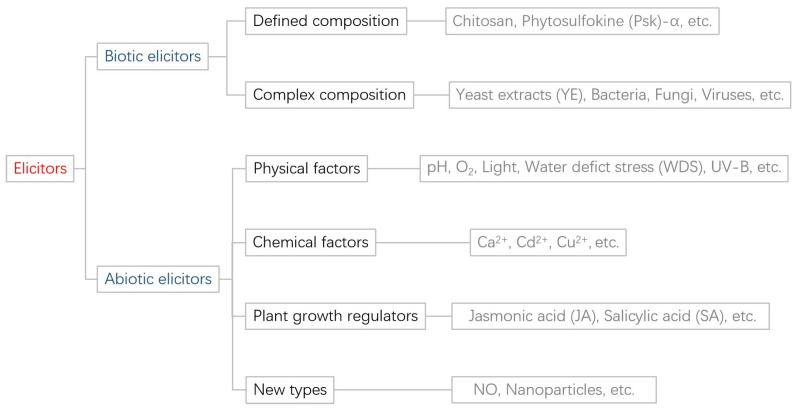
Elicitor classification diagram.

Elicitors are molecules that are typically generated by pathogens, insects, or other organisms. These molecules are recognized by specific receptors in plants, leading to the activation of plant immune responses against these external threats [7]. From a pathogenesis perspective, most elicitors serve as non-toxic factors that contribute to the development of genetic pseudo-resistance in plant innate immunity. These elicitors are recognized by R proteins or plant receptors localized at the cytoplasm or plasma membrane to initiate signaling pathways, leading to immune responses and the biosynthesis of secondary metabolites [8]. The signal transduction mechanism can be summarized as follows: the specific assembly of the elicitor with the receptor causes the conformational change of the receptor or the activation of receptor kinase. This, in turn, indirectly activates its corresponding effectors, such as G proteins, ion channels, and lipases, which then transduce the signal further downstream to initiate an immune response. The precise intricacies of the mechanism remain uncertain. Plant-specific recognition of elicitors provides a comprehensive explanation for the selective ability of certain elicitors to induce the accumulation of phytochemicals or secondary metabolic compounds in specific plant species [9]. The elicitor signal is a multi-component network with a variety of responses in succession, consisting of multiple parallel or cross-linked signaling pathways that lead to different targeting responses and may change with the recognition of different elicitor signals [10]. Elicitors include biotic elicitors, such as bacteria, fungi, viruses, and plant cell wall components, as well as abiotic elicitors, including environmental factors, metal ions, and hormones [8] (Figure 2). In addition to being classified based on their source, elicitors can also be classified based on their chemical properties, structure, and other factors. It is worth noting that certain elicitors may fall into multiple categories simultaneously.

Many factors can influence the elicitation effect, such as the choice of the elicitor and elicited plants, the concentration, duration, and site of the elicitation, and the composition of the medium [11]. Elicitation can be carried out in vitro or in vivo in plants. In vitro, elicitation mainly occurs in cultured cells, organs, or tissues. In vitro elicitation enables faster product synthesis compared to whole-plant culture [12]. Although studies have shown that the elicitation of plant cell cultures can effectively enhance the production and accumulation of valuable secondary metabolites [13], the instability and variability of this method make it difficult to apply in production practice [14]. By way of contrast, hairy roots are considered to be the material for elicitation studies due to their rapid growth, suitability for large-scale culture, genetic and biochemical stability, and ability to produce similar or higher levels of alkaloids compared to intact plants [15,16]. When plant tissue is infected with *Agrobacterium rhizogenes* carrying the Ri plasmid, the infected areas will grow adventitious roots. These roots can then be cut off and grown as individual clonal lines in hormone-free solid or liquid media that contain essential nutrients. This results in the formation of hairy root systems [17,18]. Additionally, in vivo induction experiments are also conducted using foliar spraying, root watering, or seed soaking of plant bodies. The elicitation strategy has been widely used to promote the accumulation of secondary metabolites, showing promising applications. In addition to its use in plants that produce TAs, it has also been utilized in a range of medicinal plants, including *Panax ginseng*, *Calendula officinalis*, and *Digitalis purpurea* [19,20,21]. By promoting the accumulation and yield of secondary metabolites, the elicitation strategy can effectively reduce the production cost of related drugs. This is valuable for addressing the issue of a shortage of natural medicine resources.

## 3. Effects of Biotic Elicitors on TAs

### 3.1. Sugars, Proteins, and Their Precursors and Derivatives

According to Rothe et al., sugar is not only a carbon source but also a signaling compound for root cultures [22]. Chitosan is a non-antigenic, non-toxic, and biocompatible polysaccharide polymer derived from chitin [23]. In recent years, it has been shown that chitosan has been widely used as an elicitor due to its favorable physicochemical properties and multidirectional biological activity [24,25]. The induction of TAs biosynthesis by chitosan varies among species. At pH 5.5 and certain concentrations, chitosan positively affects the accumulation and release of hyoscyamine and scopolamine from *Brugmansia candida* hairy root cultures (optimum concentration for accumulation is 10 mg/L and for release is 1000 mg/L) [26]. However, chitosan did not affect the accumulation and release of alkaloids in *Atropa belladonna* [27,28] and was even lethal to *Hyoscyamus niger* [29]. Oligogalacturonides, which belong to a group of oligosaccharides, are pectin fragments derived from the partial degradation of high galacturonic acid in plant cell walls [30]. It has been shown to be the most effective elicitor of plant responses in smaller pectin fragments [31]. Oligogalacturonides promoted the yield of TAs in the hairy roots of *Datura stramonium*, and a significant increase in the amount of tropine was found by testing precursor concentrations [32]. Although sucrose is not explicitly categorized as an elicitor in some literature, it is also capable of activating immune responses in defense [33,34,35]. Therefore, it is discussed here as an elicitor. Noteworthily, while the accumulation of biomass is often thought to favor the yield of the corresponding secondary metabolites, there is an exception in the production of elicitors. The relationship between biomass and alkaloid content may be modified in some way by sucrose [36]. In addition, hairy root clones from the same plant infected with different *Agrobacterium* species respond differently to the same elicitor. One established hairy root clone of *Hyoscyamus muticus*, Cairo LBA1S, grew poorly at lower sucrose concentrations but had twice the hyoscyamine content of another clone, C58A [37]. We need to explore optimal elicitation conditions for the different clones. As far as we know, the physiological and biochemical status of hairy root clones can vary, even when obtained from the same *Agrobacterium* infestation. Therefore, it is often necessary to test the selection of high-performance root systems before conducting elicitation experiments.

Protein is an important macromolecular substance in plants, and its precursors and derivatives play a key role in growth and development. Phytosulfokine (Psk)-α, a sulfated pentapeptide isolated from *Asparagus officinalis* cultures, has been identified as a promoter of cell proliferation [38,39]. Stimulation of *Atropa belladonna* hairy roots with 10^−4^ and 10^−5^ mM of Psk-α promotes the production of TAs in the roots, specifically hyoscyamine [40]. Casein hydrolysate can provide cultures with a mixture of organic nitrogen, phosphate, trace elements, vitamins, and amino acids. However, its addition may be unnecessary for growth and production when nutrients are already available in the medium. For instance, a study found that adding casein hydrolysate to *Hyoscyamus niger* root cultures grown under optimal conditions did not have a significant effect on their TAs production [29]. Pectinase is a general term for a category of enzymes that break down pectin. Its main sources are microorganisms, and it has also been studied as an elicitor in some experiments [41,42]. Pitta Alvarez et al. found that pectinase increased the hyoscyamine content in *Brugmansia candida* hairy root cells and promoted the release of both hyoscyamine and scopolamine. However, the acetate buffer was found to be superior in comparison [43]. An enzyme called hemicellulase was found to increase the levels of hyoscyamine and scopolamine in *Brugmansia candida* hairy roots at 24 and 48 h after addition, and it also facilitated the release of scopolamine [44]. Both hemicellulases and pectinases break down the components of the plant cell wall, thereby altering its mechanical strength. It is speculated that their mechanisms of action may be related to this [45].

### 3.2. Yeast Extract

Yeast extract (YE) is a complex product containing different types of macromolecules and small molecules with diverse nutrients [46]. Studies have shown that YE can accelerate the synthesis and increase the accumulation of metabolites by activating the relevant enzymes in the metabolic pathway [47,48]. YE is also applied in the production of TAs. Guo et al. found that the addition of YE increased the content of metabolic precursor amino acids (ornithine and arginine) in *Atropa belladonna*. It also led to an upregulation in the expression of the rate-limiting enzyme genes *PMT*, *TRI*, and *H6H* in the synthetic pathway, thereby enhancing secondary metabolism and ultimately increasing the yield of hyoscyamine and scopolamine [49]. Hedayati et al. used different concentrations of YE (0, 0.5, 1, and 1.5 mg/L) as an elicitor to treat the hairy roots of *Atropa belladonna*. The results showed that the highest level of scopolamine and atropine yield was achieved under the treatment of 1 mg/L and 1.5 mg/L of YE for 48 h, respectively [50]. YE also increased the levels of hyoscyamine and scopolamine in *Brugmansia candida* hairy root cells, and more importantly, the ratio of scopolamine content to hyoscyamine content (St/Ht) was significantly increased [51]. Scopolamine has a higher value than hyoscyamine, the precursor of scopolamine, due to its fewer side effects, better efficacy in medicinal applications, and low abundance in many species [52,53]. The increase in this ratio is partly an indication of the value of YE in scopolamine production.

### 3.3. Fungi

Fungi are composed of a variety of complex compounds, including chitin, dextran, NEP1-like protein (NLP), Harpin protein, and many other proteins, as well as various secreted proteins, all of which can be used individually as an elicitor [54]. Current research primarily focuses on the antimicrobial activity of TAs and the changes in substances following the feeding of TAs [55,56,57]. In fact, fungi also have an influence on the synthesis and accumulation of TAs. Adding cell wall fragments of *Phytophthora megasperma* (Pmg) to *Datura stramonium* cell cultures increased the final production of TAs five-fold, as reported by Ballica et al. [58]. Three fungi (*Aspergillus niger*, *Alternaria* sp., and *Fusarium monoliforme*) effectively promoted the accumulation of hyoscyamine and scopolamine despite inhibiting the growth of *Datura metel* hairy roots. Among these fungi, *Aspergillus niger* was found to be the most effective elicitor [59]. Pitta Alvarez et al. cultured the hormone-like fungus *Hormonema* ssp. isolated from *Brugmansia candida* in two different substrates, Sabouraud-dextrose and MSRT, and treated the hairy roots of *Brugmansia candida* separately with the fungal homogenate and medium. The accumulation and release of hyoscyamine and scopolamine varied significantly with treatment time. The authors speculate that due to the complex composition of the fungal homogenate and the spent medium, multiple elicitors may be induced to act together on the accumulation and release of TAs [43].

### 3.4. Bacteria

Compared to fungi, bacterial elicitors have good aspects, such as shorter culture times and simpler preparation [60]. One study used two Gram-positive strains (*Staphylococcus aureus* KCTC 1916 and *Pseudomonas aeruginosa* KCTC 1750) and one Gram-negative strain (*Bacillus cereus* KCTC 1012) to induce *Scopolia parviflora* hairy roots. The first two were found to be more effective than the latter in the production of scopolamine, and they all increased the St/Ht ratio in the roots, especially *Staphylococcus aureus* KCTC 1916. This would also imply that the three strains may have increased the conversion of hyoscyamine to scopolamine. However, paradoxically, the expression of *H6H* was reduced, suggesting that other unknown regulatory pathways may exist [61]. Moussous et al. explored the influence of four *Pseudomonas* strains (*Pseudomonas putida* PP01 and *Pseudomonas fluorescens* P64, P66, and C7R12) on the levels of TAs in three *Datura* transgenic root lines (*Datura stramonium*, *Datura tatula*, and *Datura innoxia*). The results indicated that several of the *Pseudomonas* lines studied could positively and significantly affect hyoscyamine and scopolamine. The highest levels of hyoscyamine were observed in *Datura tatula* lines exposed to C7R12 for 5 days, while scopolamine levels were highest in *Datura innoxia* lines exposed to P64 for 5 days, and P66 for 5 days and 10 days [62].

### 3.5. Viruses

Viruses, as foreign invaders, interact with the plant body and cause corresponding changes in secondary metabolism [63]. Different classes of secondary metabolites have also been shown to be resistant to pathogens [64]. Examples of viral infections to increase specific secondary metabolites are rare, likely because their mechanism of action is more intricate compared to standard biotic or abiotic elicitors. Mihálik et al. used three tobacco mosaic viruses (PMMoV, TMV, and ToMV) to artificially infect *Datura stramonium* through in vivo elicitation. They discovered that the hyoscyamine content was significantly higher compared to in vitro induction or non-elicited capillary root cultures, which illustrates the potential value of viral elicitation [65]. In order to clearly demonstrate the role of each biotic elicitor in TAs biosynthesis, a table (Table 1) is provided, which includes all the relevant elements. Some of these elements are not described in detail in the main text. The rising arrow in the table represents a positive impact, and the falling arrow represents a negative impact.

## 4. Effects of Abiotic Elicitors on TAs

### 4.1. Physical Elicitors

Physical elicitors include factors related to the environment. Several studies have demonstrated that the release of TAs is facilitated by an appropriate reduction in the pH of the medium. An elevated release of hyoscyamine and scopolamine was observed in *Brugmansia candida* hairy roots when subjected to specific concentrations of citric acid and acetic acid at different growth stages [26]. According to the alkaloid “ion trapping” mechanism, alkaloids tend to be mobile and stored in media with low pH [68]. Acetic acid molecules have the potential to enter the TAs synthesis pathway through conversion to acetoacetyl-CoA, and citric acid may act by influencing the TCA cycle [26,43]. Acids can also change the properties of cell walls and cell membranes, thereby affecting the release of alkaloids [69,70]. In conclusion, there are a number of reasons that could be used to explain this phenomenon. Oxygen is necessary for both plant growth and the biosynthesis of TAs [71]. Under conditions of pure oxygen, the root cultures of *Duboisia myoporoides* exhibited elevated levels of H6H and tropine. This led to the activation of the scopolamine synthesis pathway while simultaneously inhibiting the synthesis pathway of nicotine and other tropine derivatives. Consequently, the production of scopolamine was facilitated [72]. In the conventional hairy root bioreactor, little vigorous mixing is employed to prevent harm to the root system, which, however, leads to inadequate oxygen provision [73]. The objective of reactor development is to achieve a reasonable and effective supply of oxygen. Light makes a significant impact on plant growth and production of TAs. *Hyoscyamus albus* root cultures showed an increase in alkaloid content under light, especially scopolamine [74]. However, for the roots of *Atropa belladonna*, the effect of light on scopolamine and calystegine contents was almost the same, and scopolamine was only present in trace amounts [22]. Plants exposed to stressful growth conditions are likely to slow down their metabolism in favor of accelerated synthesis of non-toxic alkaloids, potentially serving as a form of nitrogen storage [75]. Drought, one of the major stressors in physical conditions, causes a slowdown in growth but can mostly positively affect the synthesis of active compounds in medicinal plants. *Hyoscyamus muticus* hairy roots were subjected to osmotic stress treatment using mannitol to simulate water stress. The results revealed a decrease in the biomass of the hairy roots, while the total production of hyoscyamine increased twofold [76]. The content of hyoscyamine and scopolamine in *Atropa belladonna* hairy roots increased significantly under high water deficit stress (WDS) and high nitrogen fertilization [75]. UV-B radiation, as one of the stresses, was able to stimulate the synthesis of secondary metabolites in different organisms [77]. UV-B stress exerted on hairy root cultures of *Anisodus luridus* demonstrated significant up-regulation of four genes, *PMT*, *TRI*, *CYP80F1*, and *H6H*. Additionally, it was observed that the levels of hyoscyamine decreased while scopolamine levels increased due to the facilitated conversion of hyoscyamine to scopolamine, with no effect of UV-B on the release of either [78].

### 4.2. Chemical Elicitors

Chemical elicitors are commonly linked to a range of ions. Ca^2+^ is a well-established second messenger that produces a marked effect on signal transduction and cellular regulation [79]. It can also induce defense responses, which is similar to the mechanism of action exhibited by elicitors in general [80]. It has been shown that Ca^2+^ activates the expression of *PMT* in the synthetic pathway, thereby enhancing the production of TAs [81]. Gontier et al. discovered that adding 10 mM CaCl_2_ to suspension cell cultures of *Datura innoxia* led to an approximately tenfold increase in the amount of hyoscyamine and scopolamine in the cells [82,83]. The study conducted by Boualem found that the treatment of approximately 9 mM CaCl_2_ for 24 h resulted in the highest yield of TAs in *Datura innoxia* hairy roots [84]. For the hairy roots of *Datura stramonium*, higher concentrations of Ca^2+^ (~18 mM) significantly increased hyoscyamine content, while lower concentrations of Ca^2+^ (less than 1 mM) inhibited PMT activity, resulting in a decrease in hyoscyamine content [84,85]. Al is a silvery-white light metal like Ca monomers. Micromolar levels of Al-induced in micropropagated plants of *Datura innoxia* were found to promote the activity of the antioxidant enzyme, scavenging of ROS, prevention of oxidative damage, and an increase in the content of TAs [86]. Heavy metal ions will disrupt the structure of the cytoplasmic membrane and increase the permeability of substances, which are detrimental to the integrity and viability of plant tissues [87]. CdCl_2_ and CuCl_2_ have adverse effects on the growth of *Atropa belladonna*, *Brugmansia candida*, and *Datura stramonium* hairy roots while promoting the release of hyoscyamine and scopolamine [27,51,88]. Certain heavy metal ions have the potential to induce the synthesis and accumulation of TAs. The presence of the Ag+ in *Anisodus acutangulus* resulted in an elevation of putrescine levels and the expression of *AaPMT1*, with a trend of increasing, followed by decreasing, and then increasing production of TAs up to 96 h compared to the control [89]. In vitro propagated *Atropa belladonna* plants elicited with chromium revealed increased levels of *H6H* transcripts and elevated levels of hyoscyamine and scopolamine [90].

### 4.3. Plant Hormones and Growth Regulators

The main plant hormones include auxin (IAA), gibberellin (GA), jasmonic acid (JA), cytokinin (CTK), ethylene(ETH), abscisic acid (ABA), brassinosteroids (BR), salicylic acid (SA), and strigolactone (SL) [91]. In addition to these, there are many other plant growth regulators and their derivatives with similar effects. Among them, the JA and SA analogs are most studied for their ability to induce TAs. The following section highlights the actions of these two substances.

JA is an important signal for the biosynthesis of many plant secondary metabolites, and JA signaling is a significant pathway that regulates the induced systemic resistance (ISR) mediated by inter-rhizosphere bacteria [92]. Methyl jasmonate (MeJA), the methyl ester of JA, is widely recognized as a potent elicitor for inducing the accumulation of TAs in Solanaceae. It has been shown to promote the accumulation of TAs in the hairy roots of *Anisodus acutangulus*, *Atropa baetica*, *Scopolia parviflora*, *Hyoscyamus niger*, and others. The genes involved in the biosynthesis pathways of TAs in various species were found to be more responsive to MeJA. Analysis of gene expression profiles revealed that *TRI* expression was increased in *Anisodus acutangulus*, *PMT* and *H6H* expression was increased in *Atropa belladonna*, MeJA may transiently regulate *PMT* and *H6H* expression in *Scopolia parviflora* under MeJA treatment [89,93,94]. MeJA can effectively enhance the activity of endogenous H6H in *Hyoscyamus niger*, thereby facilitating the conversion of hyoscyamine to scopolamine and significantly augmenting the economic value [95]. In contrast, a separate elicitation experiment conducted on the *Atropa belladonna* hairy roots yielded contrasting findings. As the concentration of MeJA increased, there was an observed enhancement in *H6H* expression, but accompanied by an increase in hyoscyamine content and a decrease in scopolamine content. This outcome can be attributed to the increase in the substrate hyoscyamine but a limited amount of H6H and inhibition of H6H enzyme activity [93]. Similarly, the induction of *Hyoscyamus muticus* root cultures by JA resulted in the accumulation of synthetic precursors (putrescine, methyl putrescine), but the production of hyoscyamine and scopolamine was not effectively induced [96]. This also suggests that increasing precursor mass does not necessarily result in an appreciable increase in end-product synthesis, which may involve extremely complex regulatory mechanisms. In addition to stimulating the synthesis of TAs in root cultures, MeJA can also act as an osmotic agent to facilitate the accumulation of substances in the medium [96]. Jaremicz et al. treated hairy roots of *Hyoscyamus niger* with 0.1 mM and 1 mM of MeJA, respectively, and found that the medium hyoscyamine and scopolamine content was higher than the control at the time of the assay, especially at the 1 mM treatment [53]. Similar results were obtained for the elicitation of *Datura stramonium* hairy roots using MeJA, which is highly advantageous due to the relatively easier collection of extracellular alkaloids [97].

SA mainly mediates systemic acquired resistance (SAR), and its increased levels are often seen as a marker of SAR [98]. There is a strong correlation between increased levels of SA and its conjugates in infected plants and the development of disease resistance [99]. This also suggests that SA might be a preferable elicitor. The effect of SA and its derivative acetylsalicylic acid (ASA) on secondary metabolites exhibits variability across different species. SA increases scopolamine levels in *Scopolia parviflora* adventitious root cultures by inducing the expression of *H6H* [94]. Harfi et al. elicited three *Datura* species (*Datura stramonium*, *Datura tatula*, and *Datura innoxia*) with SA and ASA, then found that 0.1 mM was the optimum treatment concentration for all three *Datura* species, with the highest hyoscyamine yield of all treatments obtained at 0.1 mM ASA for *Datura tatula* [100]. *Anisodus luridus* hairy root cultures were induced with three different concentrations (0.01 mM, 0.1 mM, and 1 mM) of ASA. The results revealed that 1 mM ASA resulted in the highest expression levels of *PMT*, *TRI*, *CYP80F1*, and *H6H*, corresponding to the strongest TAs synthesis and significantly induced the release of scopolamine [78]. In contrast, the induction of *Anisodus acutangulus* hairy roots with SA dissolved in ethanol revealed a decrease in the average production of hyoscyamine and scopolamine but a consistent increase in the production of anisodine. Interestingly, when treated with ethanol alone, it was observed that the expression of *H6H* increased, the competition response was inhibited, and the yield of TAs effectively increased. This suggests that SA may strongly inhibit the effect of ethanol during the synthesis of TAs in *Anisodus acutangulus* [89]. In a study conducted, SA (0.2–2 mM) had no significant effect or even a negative effect on TAs accumulation in *Atropa belladonna* hairy roots, but the release ratio of TAs increased as the concentration of SA exceeded 0.5 mM. It showed that SA possesses a strong capability in facilitating the release of TAs [101].

In addition to the aforementioned commonly used hormones, researchers have explored the impacts of various other prevalent hormones. IAA and its analogs are usually detrimental to the synthesis of TAs in hairy roots. Probably because the T-DNA of *Agrobacterium tumefaciens* contains the gene responsible for IAA synthesis, the addition of exogenous IAA-like substances causes excessive inhibition [102]. This seems to be confirmed by the decreased production of TAs in *Hyoscyamus niger* and *Datura stramonium* when the external concentration of IAA analogs is increased [103,104,105]. However, there are exceptional cases, as observed in *Hyoscyamus muticus*, where the authors postulate the possibility of a deficiency in endogenous IAA within the cultured root system [102]. GA and ABA are among the main internal signals for plant survival and growth in stressful environments [106]. There are at least 37 known species of GA. GA_7_ can positively affect the accumulation and transformation of TAs in *Brugmansia candida* at effective concentrations of 10^−4^, 10^−1^, 1 mg/L, and 10^−1^ mg/L for two different clones [107]. Both GA_3_ and ABA strongly inhibited the production of scopolamine in the hairy roots of *Hyoscyamus muticus* but had no significant effect on root morphology [102]. ABA did not alter the content of TAs in *Datura stramonium* root cultures, but in another study, ABA promoted alkaloid accumulation in leaves, suggesting the conjecture that ABA-related receptors may be in the leaves [105]. However, this does not explain well the results obtained in the former plant. Different effects of ABA on the TAs content of leaves and roots were indeed also found in studies on the effect of ABA on TAs content in *Anisodus acutangulus* plants. In the 24 h period, the roots exhibited an increase in hyoscyamine and anisodine content, while scopolamine content decreased. Meanwhile, the leaves showed an increase in scopolamine and anisodine content, with a decrease in hyoscyamine content. Surprisingly, none of the genes in the synthesis pathway were significantly induced, The reason has not been clearly explained [108].

In recent years, new plant growth regulators have been discovered and applied in the production of TAs as elicitors. Glyphosate belongs to the organophosphorus herbicide class and functions as a plant growth regulator [109]. It reduced the content of phenylalanine in jimsonweed (*Datura stramonium*) seedlings, reduced the content of tropinone and tropine at 10^−7^ and 10^−6^ M, as well as the expression of *PMT* mRNA transcripts in roots at 10^−6^ M. Although no direct influence on the content of hyoscyamine and scopolamine was revealed, a significant inhibitory effect can be inferred [110]. Coronarin (COR) is a bacterial toxin produced by *Pseudomonas syringae* [111]. The mechanism of action of COR is to mimic a bioactive JA coupling (JA-Ile), which subsequently targets the JA receptor for additional modulation [112]. Its effect on the synthesis of TAs is more complex. The inhibitory effect of COR at 0.5 uM on hyoscyamine production in the hairy roots of *Atropa acuminata* increased with time, but scopolamine levels were found to be fivefold higher than the control at one week after treatment [113].

### 4.4. New Types

Nanoparticles are widely used in medicine and immunology-related fields and, in recent years, have emerged as a novel inducing material capable of eliciting metabolic and physiological responses [114]. They can increase the activity of nitrate reductase and glutamate dehydrogenase to affect nitrogen metabolism in plants, thus increasing protein levels, stimulating gene expression, and inducing the biosynthesis of secondary metabolites [115]. Different researchers have made attempts to apply nanoparticles in the production of TAs. Moharrami et al. treated the hairy roots of *Hyoscyamus reticulatus* with different concentrations of iron oxide nanoparticles and found that the maximum levels of hyoscyamine and scopolamine were reached at 900 mg/L for 24 h and 450 mg/L for 48 h, respectively. The genetic DNA experienced toxicity as a result of higher concentrations and longer treatment times, leading to a decrease in product content [116,117]. The nanoparticles could provide abundant Fe^2+^ for the enzymatic reaction, and further analysis showed that this induction method had an effect on the activity and expression of both *PMT* and *H6H*, thus increasing the yield of the corresponding TAs [118]. Iron oxide nanoparticles could also stimulatingly affect the expression of *H6H* in *Atropa belladonna* hairy roots, leading to the accumulation of scopolamine [119]. Similarly, the application of different concentrations of zinc oxide nanoparticles (50, 100, 200, and 24 mg/L) to *Hyoscyamus reticulatus* hairy roots led to the highest levels of hyoscyamine and scopolamine content at 100 mg/L, 48 h, and 100 mg/L, 72 h, respectively. The analysis using RT-PCR demonstrated that zinc oxide increased the expression level of the *H6H* transcript, and scopolamine accumulation was positively correlated with *H6H* expression [115]. 100 mg/L of silica nanoparticles treated with *Hyoscyamus reticulatus* for 24 h revealed that the highest levels of hyoscyamine and scopolamine were achieved through the increase of *PMT* and *H6H* expression levels [120].

Sodium nitroprusside (SNP) is a nitric oxide donor that releases NO, which is involved in disease and stress resistance responses in plants as a cellular and intercellular signal molecule [121]. NO can interact with JA, MeJA, and SA signals to mediate the biosynthesis of secondary metabolites [122]. Treatment of *Hyoscyamus reticulatus* hairy root cultures with various concentrations of SNP resulted in significant alterations in the activities of antioxidant enzymes, including ascorbate peroxidase (APX), catalase (CAT), and peroxidase (POD). Additionally, the production of hyoscyamine and scopolamine reached a maximum at 50 μM, 48 h and 100 μM, 24 h, respectively [123]. The effects of abiotic elicitors on TAs are presented in Table 2, including some that are not extensively discussed in the main text.

## 5. Effects of Combined Elicitation on TAs

It is a common practice to employ multiple elicitors to investigate their impact on the yield of TAs. Since several elicitors have a positive effect on TAs biosynthesis, it is worthwhile to explore the potential synergistic effects that may arise from their combined application. Additionally, if one elicitor produces a negative impact, we also need to assess the potential of other elicitors to counteract or alleviate adverse effects, particularly in relation to physical stress responses.

We have previously described the respective effects of different elicitors. In the following section, we will describe the combined effects of various elicitors. Cyclodextrins (CDs) are a class of cyclic oligosaccharides produced by *Bacillus* that have the ability to induce immune responses and promote the accumulation of secondary metabolites in plants [129,130]. Co-treatment of *Atropa acuminata* hairy roots with 50 mM methyl-β-cyclodextrin (β-CD) and 0.5 uM of COR was found to positively affect both scopolamine production and hyoscyamine release. However, the same treatment negatively affected both hyoscyamine and scopolamine production from *Atropa belladonna* hairy roots [113]. Ghorbanpor et al. discovered that the combination of biotic elicitors (plant growth promoting rhizobacteria (PGPR) strains) and abiotic elicitors (WDS) on *Hyoscyamus niger* plants was very beneficial. At low WDS levels (30% depletion in field water holding), *Pseudomonas putida* (PP) is considered effective, while at moderate and heavy levels (60% depletion and 90% depletion in field water holding), *Pseudomonas fluorescens* (PF) is considered more effective [131]. Studies have shown that PGPR can improve the activity of related enzymes in plants through the release of plant growth regulators, and WDS also induces the yield of IAA in plants, with a clear synergistic amplification between the two in this respect [132]. Khanam et al. discovered that a combination of two growth factors and CTK(10 mM benzyladenine (BA) + 1 mM napthaleneacetic acid (NAA) and 10 mM BA + 0.1 mM indolyl-3-butyric acid (IBA)) in cultured *Duboisia myoporoides* rootless shoots also produced TAs [133], in contrast to some species without TAs in rootless shoots [134,135]. This greatly breaks our existing understanding, as to our knowledge, TAs are primarily produced in the roots, and this culture provides us with a novel avenue for further investigation. The authors also show that a further search for the optimal combination of plant growth regulators on this basis would facilitate the production of more TAs in rootless shoots.

Understanding the mechanisms of interaction between multiple elicitors is important when designing to enhance induction, as it allows for better regulation. For instance, the much-studied signaling pathway between SA and JA/ETH may respond differently under different conditions [136]. Unfortunately, there is little literature available to elucidate the mechanisms underlying the interactions between different elicitors during the biosynthesis of TAs. The effects of combined elicitors on TAs are presented in Table 3. It should be noted that certain effects are not extensively discussed in the main text.

## 6. Combination of Elicitors with Other Strategies to Increase TAs

In addition to elicitation, strategies such as gene overexpression, transcription factor regulation, plant polyploidization, and precursor feeding are also effective methods to improve the synthesis of secondary metabolites in medicinal plants [5,138,139,140]. The combination of multiple strategies tends to improve the content and/or yield of plant secondary metabolites more effectively compared to the single strategy. The literature has documented the utilization of a combination of three strategies along with elicitors (Table 4).

Overexpressing key genes involved in the synthetic pathway of TAs in plants/hairy roots is a common strategy to enhance the yield of TAs. In a scientific experiment, transgenic *Atropa baetica* hairy roots that overexpressed the *H6H* gene were induced with SA, MeJA, and ASA. It was observed that MeJA (0.1 mM, 4 h) had the most pronounced impact on the accumulation of scopolamine, with a 25-fold improvement in *H6H* gene expression [107]. Overexpression of *PMT* in *Hyoscyamus niger* hairy roots caused an increase in PMT activity and an increase in methylputrescine content but was unable to significantly increase TAs content. In contrast, PMT and H6H activity was significantly enhanced, and scopolamine content increased after MeJA treatment. Once again, this indicated the strong induced effect of MeJA on the synthesis pathway of TAs [99]. For medicinal plants, polyploids are often more valuable for the higher biomass and bioactive compounds in comparison to haploid plants [143]. Belabbassi et al. induced *Datura stramonium* hairy roots with different concentrations of colchicine to obtain tetraploid hairy root systems. After SA and ASA treatments, a notable increase in the yield of scopolamine was observed in comparison to the other treatment groups and control groups. This finding suggests the favorable combined effect of polyploidization and elicitation on the synthesis of scopolamine [141]. The precursor feeding strategy is an efficacious approach to enhancing the yield of secondary metabolites in plants, as the endogenous level of biological precursors is usually a major limiting factor for biosynthesis. Boitel et al. discovered that the addition of the surfactant Tween 20 and the precursors (L-phenylalanine or DL-β-phenyllactic acid (0.5 mM)) at the late growth stage of *Datura innoxia* hairy roots greatly increased the total hyoscyamine content, whereas feeding the precursors alone had no effect on the synthesis of TAs. Tween 20 was characterized as a chemical elicitor that potentially possesses the capability to induce the synthesis pathway and the release of TAs [142]. This provides visual experimental evidence for combining elicitation with a precursor feeding strategy to enhance TAs production.

In addition to the above-combined strategies, the combination of immobilization of cultured cells or in situ, product removal with elicitation treatment has not been reported to improve the metabolic yield of TAs. However, these approaches have been applied in *Plumbago indica*, *Tripterygium wilfordii*, and *Lithospermum erythrorhiz*, resulting in increased production of secondary metabolites [144,145,146]. In the future, the combination of these two strategies can also be applied to medicinal plants of the Solanaceae family in order to offer novel techniques for enhancing the synthesis of TAs.

## 7. Prosperity

Previous studies have shown that the utilization of various elicitors, either individually or in combination, along with other strategies, effectively regulates the content change of TAs in medicinal plants. With the change in elicitation concentration and experimental duration, the regulatory effect may be complex and variable. Simultaneously, achieving identical outcomes by using the same concentration of elicitors in the same plant is challenging due to variations in external conditions and the diverse physiological and biochemical states of plant lines. Therefore, we emphasize that the existing data should only be used for reference, and it is necessary to further optimize the elicitation conditions based on the actual situation. In the elicitation studies of TAs, hairy roots are the predominant site for elicitation. Hairy root elicitation is an optimal model for fundamental research in the commercial production of TAs. There are currently very limited examples of this strategy in industrial practice, hampered by the immaturity of large-scale cultivation techniques for hairy roots. We believe that the utilization of automated technology to regulate multiple parameters within the hairy root bioreactor and complete the extraction of TAs can effectively overcome this constraint and expedite the commercialization process.

In the exploration of the elicitation mechanism, due to the underdeveloped state of technology development and the ambiguity of genes related to TAs synthesis, the previous experiments were rarely able to show the comprehensive gene expression and transcriptional regulation change during induction. With the complete elucidation of the TAs biosynthesis pathway, an increasing number of researchers have attempted to explain the elicited changes at the molecular level with some success. However, the signal transduction pathway involved in the elicitor treatment is not a monolithic and linear process. Instead, it comprises a tightly linked network of numerous genes and transcription factors, which also makes it extremely difficult to completely elucidate the mechanism of action of the elicitor at the molecular level. In addition to employing conventional methods for transcriptional regulation analysis, researchers have endeavored to use mathematical modeling and algorithmic prediction to address pertinent issues. As a result, substantial advancements have been achieved in many research domains, including disease intervention and organ development. We possess grounds to assert that information technology will also play an increasingly important role in the study of elicitors. At the same time, relevant procedures need to be further developed and improved to achieve the goal of accurate prediction. Based on an analysis of the relevant mechanisms, the combination of joint elicitation and multi-strategy approaches will further stimulate the production potential. Additionally, the precise regulation of metabolic pathways will offer greater opportunities for the identification of novel elicitors, thereby facilitating the synthesis of TAs.

## Figures and Tables

**Table 1:** Effects of biotic elicitors on TAs.

Elicitor		Host Plant	Elicitation Tissue	Effect		Reference
				Hyoscyamine	Scopolamine	
Sugar, protein, and their precursor and derivative	Chitosan	*Brugmansia candida*	Hairy roots	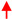	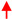	[26]
		*Atropa* *belladonna*		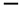	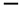	[27,28]
		*Hyoscyamus niger*		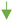	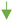	[29]
	Oligogalacturonides	*Datura* *stramonium*		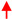	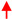	[32]
	Sucrose	*Hyoscyamus muticus*		Complex		[37]
	Psk-α	*Atropa* *belladonna*		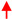	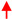	[40]
	Casein hydrolysate	*Hyoscyamus niger*		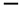	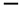	[29]
	Pectinase	*Brugmansia candida*		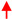	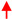	[43]
	Hemicellulase	*Brugmansia candida*		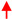	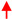	[44]
YE	YE	*Atropa* *belladonna*		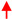	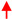	[49]
		*Brugmansia candida*		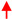	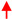	[51]
		*Hyoscyamus niger*		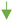	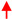	[29]
		*Datura* *metel*		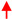	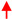	[59]
Fungi	*Aspergillus niger*	*Datura* *metel*		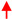	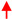	[59]
	*Alternaria* sp.			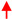	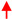	[59]
	*Fusarium* *monoliforme*			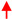	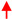	[59]
	*Hormonema* ssp.	*Brugmansia candida*		Complex	Complex	[43]
Bacteria	*Bacillus cereus* KCTC 1012	*Scopolia* *parviflora*			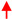	[61]
	*Staphylococcus* *Aureus* KCTC 1916 and 1750				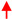	[61]
	*Pseudomonas putida* PP01	*Datura* *stramonium, Datura* *tatula*, and *Datura innoxia*		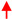	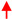	[62]
	*Pseudomonas * *fluorescens* P64, P66, and C7R12			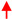	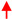	[62]
	*Staphylococus * *aureuse*	*Scopolia * *parviflor*		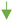	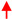	[66]
	*Bacillus cereus*	*Datura * *metel*			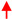	[67]
	*Staphylococcus* *aureus*				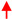	[67]
Virus	PMMoV, TMV, and ToMV	*Datura* *stramonium*	Plants	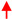		[65]
Fungi	*Phytophthora megasperma*	*Datura * *stramonium*	Cell cultures	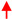	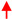	[58]

The rising arrow in the table represents a positive impact, and the falling arrow represents a negative impact.

**Table 2:** Effects of abiotic elicitors on TAs.

Elicitor		Host Plant	Elicitation Tissue	Effect		Reference
				Hyoscyamine	Scopolamine	
Physical elicitor	Acetic acid	*Brugmansia candida*	Hairy roots	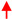	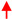	[26]
	Citric acid			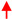	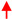	[26]
	Oxygen	*Duboisia * *myoporoides*		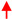	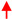	[72,124]
	Light	*Hyoscyamus albus*			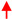	[74]
		*Datura * *innoxia*		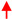		[125]
		*Atropa * *belladonna*		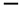		[22]
	WDS	*Hyoscyamus muticus*		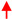		[76]
		*Atropa * *belladonna*		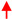	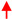	[75]
	UV-B	*Anisodus * *luridus*		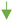	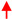	[78]
		*Hyoscyamus reticulatus*		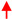	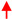	[126]
Chemical elicitor	Ca^2+^	*Datura * *innoxia*	Cell cultures	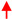	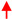	[82]
		*Brugmansia candida*	Hairy roots	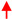	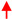	[44]
		*Datura* *stramonium*		Complex		[84,85]
	Al	*Datura * *innoxia*	Plants	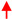	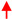	[86]
	CdCl_2_	*Atropa * *belladonna* and *Datura stramonium*	Hairy roots	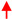	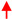	[27]
	CuCl_2_			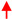	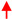	[88]
	CdCl_2_	*Brugmansia candida*		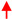	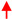	[51]
	Ag^+^	*Anisodus acutangulus*		Complex	Complex	[89]
	Cr	*Atropa * *belladonna*	Plants	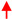	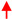	[90]
Plant hormone and growth regulator	Methyl jasmonate (MeJA)	*Anisodus acutangulus*	Hairy roots	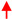	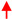	[89]
		*Atropa * *belladonna*		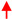	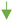	[93]
		*Scopolia * *parviflora*		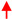	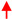	[94]
		*Hyoscyamus niger*		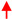	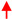	[53,95]
		*Datura * *stramonium*		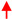	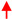	[32,97]
	JA	*Hyoscyamus muticus*		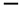	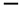	[96]
	SA	*Scopolia * *parviflora*			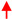	[94]
		*Brugmansia candida*		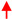	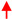	[99]
		*Anisodus acutangulus*		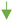	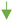	[89]
		*Atropa * *belladonna*		Complex	Complex	[101]
		*Atropa * *baetica*			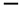	[127]
	Acetylsalicylic acid (ASA)	*Anisodus * *luridus*		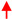	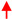	[78]
		*Atropa * *baetica*			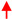	[127]
	SA	*Datura* *stramnium, Datura tatula* and *Datura innoxia*		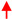		[100]
	ASA			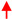		[100]
	Indole butyric acid (IBA)	*Hyoscyamus niger*		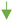	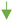	[103]
	Naphtalic acetic acid (NAA)	*Hyoscyamus niger*	Cell cultures	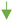		[104]
	NAA	*Datura * *stramonlum*	Hairy roots	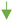	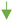	[105]
	2,4-Dichlorophenoxyacetic acid (2,4-D)			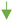	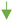	[105]
	Auxin (IAA)	*Hyoscyamus muticus*		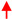	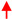	[102]
	NAA			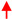	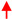	[102]
	Gibberellin A7 (GA_7_)	*Brugmansia candida*		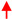	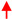	[107]
	GA_3_	*Hyoscyamus muticus*		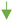		[102]
	Abscisic acid (ABA)			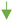		[102]
	ABA	*Datura* *stramonlum*		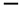	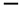	[105]
		*Anisodus acutangulus*	Plants	Complex	Complex	[108]
	Glyphosate	*Datura * *stramonium*	Seedings	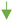	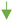	[110]
	Coronarin (COR)	*Atropa * *acuminata*	Hairy roots	Complex	Complex	[113]
New type	Iron oxide nanoparticles	*Hyoscyamus reticulatus*		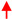	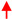	[118]
		*Atropa * *belladonna*			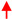	[119]
	Zinc oxide nanoparticles	*Hyoscyamus reticulatus*		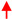	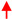	[115]
	Silicon dioxide nanoparticles			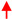	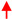	[120]
	Titanium dioxide nanoparticles	*Hyoscyamus niger*	Plants	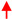	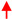	[128]
	NO	*Hyoscyamus reticulatus*	Hairy roots	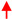	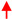	[123]

The rising arrow in the table represents a positive impact, and the falling arrow represents a negative impact.

**Table 3:** Effect of combined elicitation on TAs.

Combined Elicitation	Host Plant	Elicitation Tissue	Effect		Reference
			Hyoscyamine	Scopolamine	
Ca^2+^ + JA	*Datura stramonium*	Hairy roots	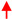		[137]
IAA + SA	*Datura metel*		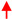		[59]
NAA + benzyladenine (BA)	*Duboisia myoporoides*	Rootless shoots	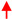	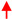	[133]
IBA + BA			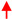	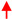	[133]
Methyl-β-cyclodextrin (β-CD) + COR	*Atropa acuminata*	Hairy roots	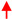	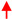	[113]
β-CD + COR	*Atropa belladonna*		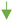	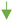	[113]
Plant growth promoting rhizobacteria (PGPR) + WDS	*Hyoscyamus niger*		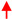	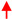	[131]

The rising arrow in the table represents a positive impact, and the falling arrow represents a negative impact.

**Table 4:** Effect of elicitation strategies in combination with other strategies on TAs.

Elicitation + Other Strategy	Host Plant	Elicitation Tissue	Effect		Reference
			Hyoscyamine	Scopolamine	
MeJA/ASA + Overexpression *H6H*	*Atropa baetica*	Hairy roots		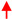	[127]
SA + Overexpression *H6H*	*Atropa baetica*			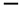	[127]
MeJA + Overexpression *PMT*	*Hyoscyamus niger*			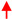	[95]
SA/ASA + Tetraploidy	*Datura stramonium*		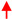		[141]
Tween 20 + L-phenylalanine/DL-β-phenyllactic acid	*Datura innoxia*		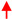		[142]

The rising arrow in the table represents a positive impact.

## Data Availability

No new data were created or analyzed in this study. Data sharing is not applicable to this article.
